# Draft Genome Sequence of Microaerobacter geothermalis Nad S1^T^, a Microaerophilic Bacterium Isolated from Tenusia Hot Spring

**DOI:** 10.1128/mra.00088-22

**Published:** 2022-04-07

**Authors:** Kian Mau Goh, Kok Jun Liew, Saleha Shahar, Iffah Izzati Zakaria, Ummirul Mukminin Kahar

**Affiliations:** a Department of Biosciences, Faculty of Science, Universiti Teknologi Malaysia, Skudai, Johor, Malaysia; b Malaysia Genome and Vaccine Institute, National Institutes of Biotechnology Malaysia, Kajang, Selangor, Malaysia; Montana State University

## Abstract

Microaerobacter geothermalis Nad S1^T^ is a rare *Bacillaceae* thermophile that grows optimally at 55°C and circumneutral pH. Although strain Nad S1^T^ was discovered >10 years ago, its genome is yet to be described. The release of the Nad S1^T^ genome sequence serves as reference genetic information for subsequent use.

## ANNOUNCEMENT

Microaerobacter geothermalis Nad S1^T^ (= DSM 22679^T^ = JCM 16213^T^) is the only described type strain of the genus *Microaerobacter* (1). The Hammam Sidi Jdidi hot spring (Nabeul, Tunisia), from which the bacterium was isolated, is located near the Mediterranean Sea (1). Strain Nad S1^T^ is an anaerobic and microaerophilic bacterium that grows optimally in 1.5 to 3.0% (wt/vol) NaCl (1). This sequencing effort aims to address the gap in type strain genome data.

The genomic DNA of Microaerobacter geothermalis Nad S1^T^ was purchased from the Leibniz Institute DSMZ-German Collection of Microorganisms and Cell Cultures GmbH (Braunschweig, Germany). A paired-end library was prepared using the NEBNext Ultra II DNA library preparation kit for Illumina (New England BioLabs, Ipswich, MA, USA), according to the manufacturer’s instructions. Sequencing was performed using the NovaSeq 6000 system (Illumina, San Diego, CA, USA) with 150-bp paired-end reads. Sequence adaptors and low-quality reads were filtered using Trimmomatic v0.39 (2). *De novo* assembly was performed using SOAPdenovo v2.40 (3), SPAdes v3.15.3 (4), and ABySS v2.3.4 (5) before integration with Contig Integrator for Sequence Assembly (CISA) v1.3 (6), and the assembly result with the smallest number of scaffolds was selected. Annotation was conducted using the NCBI Prokaryotic Genome Annotation Pipeline (PGAP) v5.30 (7). The average nucleotide identity (ANI) values for the genome of strain Nad S1^T^ versus other type species genomes were analyzed using OrthoANI v0.93.1 (8). The Genome Taxonomy Database Toolkit (GTDB-Tk) v1.7.0 was used to classify the genome (9). Default parameters were used for all software tools unless stated otherwise.

The sequencer generated a total of 1.2 Gb in 3.9 million paired-end reads. Upon removal of the low-quality reads, the assembled genome has a size of 3,132,374 bp, contributed by 85 contigs, with up to 350× coverage, an *N*_50_ value of 57,386 bp, and an average G+C content of 41.35%. A total of 3,159 genes were identified in the genome, including 3,026 protein-encoding genes, 45 pseudogenes, and 88 genes for RNA (76 tRNA, six 5S RNA, one 16S RNA, one 23S RNA, and four noncoding RNA genes). Based on the earlier study, strain Nad S1^T^ is able to use nitrate and nitrite as electron acceptors under anaerobic conditions, and the cells can reduce nitrate (1). We found multiple gene sequences for nitrite reductase, nitrate reductase subunit alpha, nitrate reductase subunit beta, nitrate reductase molybdenum cofactor assembly chaperone, and respiratory nitrate reductase subunit gamma. Nad S1^T^ transports nitrate and nitrite via its NarK family nitrate/nitrite major facilitator superfamily (MFS) transporter.

The 16S rRNA gene of Microaerobacter geothermalis Nad S1^T^ shared 90 to 92% sequence identity with those of other species affiliated with the family *Bacillaceae* (1). The genome comparison analyses indicated that the Nad S1^T^ genome shared 65.4 to 66.8% ANI with other type species genomes, such as those of Tepidibacillus fermentans, Vulcanibacillus modesticaldus, Melghiribacillus thermohalophilus, and Heyndrickxia oleronia (10–13). In addition, none of the curated genomes listed in the GTDB (9) was closely related to the Nad S1^T^ genome. The genome sequence of Microaerobacter geothermalis Nad S1^T^ will serve as reference genetic information for future research.

### Data availability.

The whole-genome shotgun sequence of Microaerobacter geothermalis Nad S1^T^ has been deposited in NCBI GenBank under BioProject accession number PRJNA797672, BioSample accession number SAMN25026507, and GenBank accession number JAKIHL000000000. The version described in this paper is the first version, JAKIHL010000000. The raw sequencing reads have been deposited in the NCBI Sequence Read Archive (SRA) with accession number SRX13800198. The 16S rRNA gene sequence of Microaerobacter geothermalis Nad S1^T^ has been deposited in NCBI GenBank with accession number FN552009.1.
